# The Reputational Consequences of Failed Replications and Wrongness Admission among Scientists

**DOI:** 10.1371/journal.pone.0143723

**Published:** 2015-12-09

**Authors:** Adam K. Fetterman, Kai Sassenberg

**Affiliations:** 1 Leibniz-Institut für Wissensmedien, Tübingen, Germany; 2 Department of Psychology, University of Essex, Colchester, Essex, United Kingdom; 3 Department of Psychology, University of Tübingen, Tübingen, Germany; Tilburg University, NETHERLANDS

## Abstract

Scientists are dedicating more attention to replication efforts. While the scientific utility of replications is unquestionable, the impact of failed replication efforts and the discussions surrounding them deserve more attention. Specifically, the debates about failed replications on social media have led to worry, in some scientists, regarding reputation. In order to gain data-informed insights into these issues, we collected data from 281 published scientists. We assessed whether scientists overestimate the negative reputational effects of a failed replication in a scenario-based study. Second, we assessed the reputational consequences of admitting wrongness (versus not) as an original scientist of an effect that has failed to replicate. Our data suggests that scientists overestimate the negative reputational impact of a hypothetical failed replication effort. We also show that admitting wrongness about a non-replicated finding is less harmful to one’s reputation than not admitting. Finally, we discovered a hint of evidence that feelings about the replication movement can be affected by whether replication efforts are aimed one’s own work versus the work of another. Given these findings, we then present potential ways forward in these discussions.

## Introduction

After years of hard work, developing studies and toiling, a scientist finally gets a pet-piece published. The scientist is proud of this accomplishment and feels good about contributing to the pursuit of knowledge. A couple years go by and people are publishing similar work and are citing the scientist’s paper. However, one day the scientist receives an email which questions the original findings. This new scientist wants to run a direct replication. The original scientist gives the replicator the materials and waits. Soon, though, other scientists and commentators on social media start talking about questionable research practices (QRPs: [1]), “p-hacking” (see [2]), and failed replications. Alongside these public discussions, the replicator has posted the results of their replication efforts. It was a failure. How should the original scientist feel?

The optimal answer is that the original scientist realizes that this is how science works and accepts the new information as insightful for future projects. Indeed, it is about the science, not the scientist. In reality, however, the common anecdotal response is worry. Scientists whose work has been targeted for replication efforts have expressed concern about their reputation as scientists [3]. This is not surprising. People fear negative evaluations and try to manage their reputations [4] and, for better or worse, scientific effects are often tied to the scientists who discover them (e.g., “Elizabeth Loftus is the car crash, memory person”). At the same time, people often overestimate the reputational consequences of self-related events and actions [5–6]. People may also take an impression management style that can backfire [7]. In this case, instead of embracing humility, the original scientist might reject the replication efforts and refuse to admit they were wrong about the effect. The current study was an attempt to test whether scientists’ reputational concerns, in the face of a failed replication effort, are warranted and whether embracing humility (i.e., wrongness admission) is a better impression management strategy than the opposite. In other words, we attempted to apply recent work on wrongness admission and impression management to the recent debates surrounding the so-called “replicability crisis” in psychology.

### Reputational Concerns and Wrongness Admission

Making a good impression is an important part of one’s social life [8–9]. People put their best foot forward and try to avoid negative evaluation. It is not controversial to state that scientists have similar concerns. Their concerns, however, might go beyond the social realm. Specifically, recent high profile instances of fraud, sloppy science, and failed replications in psychological science–and other sciences–have led to emotionally volatile debates. The emotionality underlying these debates might, at least partially, stem from reputational concerns. For example, when discussions about failed replication efforts occur in the same discussions as fraud, sloppy science, or other nefarious scientific practices, it might be hard to separate the science from the scientist; especially if it involves one’s own work. Therefore, it is understandable that a scientist might react negatively to, or reject, failed replication efforts of their work. Their reputation is, seemingly, on the line. That is, admitting to being wrong about an effect or the veracity of a failed replication effort might feel like an admission of being a poor scientist. Research, however, suggests that this should not be too strong a concern.

In the 1970s, there were a number of studies which demonstrated that being wrong in public could have a negative effect on one’s reputation. In one classic example, Ross, Amabile, and Steinmetz [10] showed that participants were judged more harshly for answering a question incorrectly, in a quiz show situation. In another set of studies, Cialdini and colleagues [11–13] found that participants were judged more harshly by onlookers if they admitted they were wrong in a brief argument. This, on the surface, suggests that being wrong and admitting it might be harmful to one’s reputation. However, Ross and colleagues’ quiz show work–on the fundamental attribution error–focused on relative judgements of the quizzer and the quizzed. And Cialdini and colleagues’ work focused on brief arguments in which the target was easily persuaded (i.e., did not put up a fight) and, in fact, the targets were actually judged more positively by the persuader.

Since the work of Cialdini and colleagues mentioned above, there has been a relative dearth of research on the consequences of public wrongness and wrongness admission. We have recently begun resurrecting this line of inquiry. Our main interest has been in showing that people overestimate the negative impact of being wrong and that admitting wrongness can have a positive effect on one’s reputation.

In one set of studies, we (Fetterman, Stolze, & Sassenberg, unpublished) investigated this idea using workplace scenarios. Participants imagined a meeting in which two characters got into a heated argument and one was clearly wrong. Some participants imagined that they were the character that was wrong and others imagined themselves as a witness to the event. All participants then rated the reputational impact for the character that was wrong. In doing so, we were able to compare the anticipated (ratings of the self) versus hypothetically actual (ratings of another) impact of this wrongness event on the character’s reputation. We predicted that participants would overestimate the negative consequences of being wrong. This prediction is in line with findings showing that people tend to overestimate the negative reputational consequences of their mistakes and foibles [6]. This can lead individuals to avoid feedback-seeking, which can be beneficial (for a review see [14]). Indeed, as predicted, reputational ratings were more negative in the condition which participants imagined the character as the self. This suggests, then, that individuals overestimate the negative impact of such an event on their reputation.

The findings of Fetterman et al. are not necessarily surprising given previous work on impression management and social biases. In fact, unrealistic concerns of negative evaluations are key features of common personality traits, such as narcissism [15] and neuroticism [16], and the spotlight effect [17]. However, we were also interested in the effects of wrongness admission, specifically, as mentioned above. Recent research suggests that humble behaviors have a relatively positive effect on reputation [18]. For example, recent studies have shown that while people are worried about appearing incompetent when seeking advice, they are actually seen as more competent for doing so [19]. Based on these findings, we (Fetterman et al.) also predicted that admitting wrongness may be good for one’s reputation. Within the same studies and scenarios described above, some participants imagined that the character (the self or other), who was wrong, admitted it; while others imagined that this did not happen. Those who imagined that the character admitted wrongness rated the character’s reputation as more positive than those who imagined that the character did not.

In addition, we found an interaction between the focus (self or other) and admission (admit or not) conditions, which indicated that while people might think that wrongness admission is bad for the reputation, it is actually best to acknowledge and admit it. Again, these findings are in line with previous work on the reputational consequences of humble behavior (e.g.,[19]), but focus on the act of wrongness admission. Fetterman and Muscanell (unpublished) further found that a person who admits when s/he is wrong in a Facebook argument is seen more positively and this leads to greater intentions to interact with that person in the future.

Taken together, our recent research in the realm of public wrongness and wrongness admission shows that people overestimate the negative reputational consequences of being wrong in public. Moreover, people think that if they admit they are wrong, others will think negatively of them, but this is not true. As such, when people see arguments on social media or the comments sections of blog posts or articles and no one wants to budge on their position, it is likely because they fear negative evaluation. This, almost certainly, can be applied to the recent “replication debates”. Original scientists of effects that have failed to replicate are defending “their” effects vehemently. They have also gone on to question replication efforts, even though they recognize the utility and necessity of these efforts. We suggest that a combination of variables is creating this response, but that reputational concerns might be one of them. Based on our previous findings, we suggest that original scientists should not need to be so concerned. However, instead of just stating this as an opinion, we sought to back it up with empirical data.

### Current Experiment

The purpose of the current experiment was to apply our recent work on wrongness admission to answer some questions surrounding the heated replication debates in psychology. We sought to do so by adapting the methods we employed in a couple of studies from Fetterman et al. Participants read a scenario dealing with a failed replication effort from the perspective of the original scientist or an outside observer. Within the scenario, the original scientist (either the self or another scientist) admits they were wrong or not. We then measured the reputational impact. We also measured participant’s feelings about the current movement toward more direct replication efforts. We designed this paradigm to answer four questions. The four questions are as follows: Do scientists overestimate the negative reputational consequences of failed replication efforts? Is admitting wrongness better or worse for one’s scientific reputation? Do scientists correctly estimate the reputational consequences of wrongness admission? And, do opinions about the so-called “replication movement” change based on whether a replication effort is targeted towards one’s own work versus the work of another scientist?

We hypothesized that 1) scientists would overestimate the negative impact of failed replication efforts, 2) wrongness admission would result in more positive reputational ratings than not admitting, 3) that scientists would not correctly estimate the reputational consequences of wrongness admission, and 4) that opinions about the so-called “replication movement” would be less supportive if replication efforts were aimed at the one’s own work versus the work of another scientist.

## Methods

### Participants and Procedures

The experiment was conducted online using Qualtrics Online Survey Solutions. We recruited participants through various forms of online communication (e.g., Twitter, Facebook, and professional email listservs) and word-of-mouth. While the channels of recruitment were primarily psychology related, participants from other fields of science were not excluded. A power analysis for a 2x2 design with the aim of detecting a medium effect size (f = .25) based on findings from Fetterman et al. (α = .05; power = .95) revealed that we needed 279 participants for adequate power. Our goal was to collect as many participants as possible in order to gain as much power as possible. However, we were drawing from a smaller population and did not expect to reach adequate levels. As such, our a priori stopping rule was that after further calls elicited no more participants, data collection was complete. Data were not looked at until the survey was closed. In all, 375 scientists clicked the experiment link. Of those, 63 participants did not complete the study, 23 had not met the requirement of having published an empirical paper, and 8 suggested that their data not be included in our analyses. This left us with 281 (107 female, 172 male, and 2 undisclosed) participants. A sensitivity analysis using G-Power indicated that this sample size allows detecting an effect with a size of f = .249 with three df and with a size of f = .216 with one df (with α = .05; power = .95). Participants had an average of 23 publications (ranging from 1 to 300), and 94 indicated that they were tenured. The participants were not compensated.

The recruitment posts and emails requested that published scientists participate in a survey about replications in science. Upon clicking the link, participants were greeted with an informed consent form. After giving consent, a disclaimer appeared that attempted to alleviate any worries of the data being used to personally attack anyone’s opinions. Participants were then randomly assigned to one of four conditions. In each condition, participants were asked to imagine a scenario (outlined below). Participants then answered a series of questions related to a target’s reputation. Demographic information, including various questions of interest, was collected and participants were thanked and debriefed. In all, the entire questionnaire was reported to take less than 5 minutes. This research was approved by the Leibniz-Institut für Wissensmedien Ethikkommission. Participants gave their consent by clicking the appropriate box on the informed consent page.

### Materials

#### The scenarios

Each participant was randomly presented with 1 of 4 scenarios. In two of the scenarios, participants were told to think about a specific finding, of their own, that they were particularly proud of (self-focused). They then read about how an independent lab had conducted a large-scale replication of that finding, but failed to replicate it. Since the replicators were not successful, they tweaked the methods and ran it again. Again, they were unable to find anything. The participants were then told that the replicators published the failed replication and blogged about it. The replicators conclusion was that the effect was likely not true and probably the result of a Type 1 error. Participants were then told to imagine that they posted on social media or a blog one of the following comments: “in light of the evidence, it looks like I was wrong about the effect” (admission) or “I am not sure about the replication study. I still think the effect is real” (no admission).

The other two conditions were highly similar. However, instead of thinking about their own work, participants were asked to think about a prominent and interesting finding that was not their own (other-focused). The rest of the scenarios were practically identical, though changed to reflect the “other-focused” nature of these conditions. In addition, the “other” scientists made comments on social media about whether they might have been wrong (admission) or not (no admission) about the original effect. These scenarios served as the 4 conditions (i.e., Self-Admission, Self-NoAdmission, Other-Admission, and Other-NoAdmission. Ns for each condition are 70, 66, 71, & 74 respectively).

#### Reputation variables

Participants responded to 15 questions with one of the above scenarios in mind. The items were designed to measure the expected (Self-Focused) versus realistic (Other-Focused) judgments of the target scientist’s reputation in response to the events that occurred in the scenario. Participants indicated their level of agreement for each item (1 = strongly disagree to 5 = strongly agree). Each item started out with either “Other scientists would…” or “I would…” depending on condition focus (i.e., Self vs. Other, respectively). Items included topics that related to the “original finding” (e.g., “….assume that I/they used questionable research practices in the initial study”), “suspicions of other work” (e.g., “….call for an investigation of my/their other work), “scientific reputation” (e.g., “….consider me/them a(s) good scientist(s)”), and “social” reputation (e.g., “….not want to collaborate with me/original authors”). Composite scores (positive items were reverse scored) were created based on these intuitive groupings in order to narrow the focus on specific types of reputation being affected. Higher scores represent more negative reputation. As these scores were substantially correlated (.62-.91), a *total* score was also created. Descriptive data and internal reliability coefficients for the total and composite scores are presented in Table 1.

**Table 1:** Descriptive Statistics and Reliability Coefficients.

* *	# items	*M*	*SD*	Alpha
**Original Finding**	4	2.58	.74	.73
**Suspicions of other work**	4	2.55	.83	.81
**Scientific Reputation**	3	2.45	.74	.78
**Social Reputation**	4	2.59	.75	.81
**Total**	15	2.54	.68	.92

**Fig 1:**
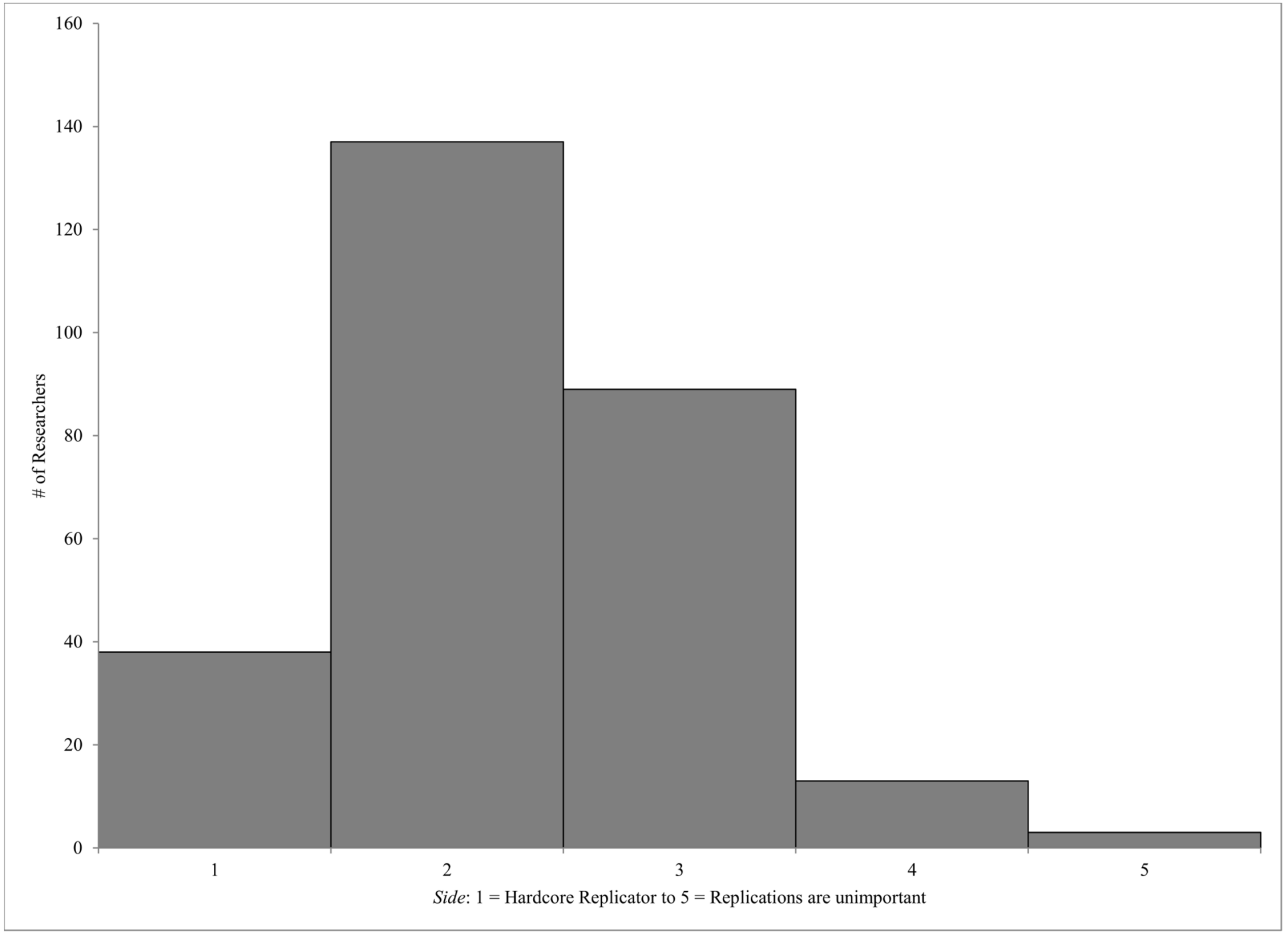
Distribution Histogram for “Side” Scores.

There were a number of other questions included in the survey. One particular question involved which “side” of the current replication movement the participant identifies with. Here, participants were to rate where they fell on the dimension of “hardcore replicator” (1) to “replications are unimportant” (5). Most people fell just to the left of center, responding with “2” (49%) or “3” (32%), with fewer falling towards the ends, and only 1% responding with “5” (See Fig 1; *M* = 2.31, *SD* = .80).

## Results

We ran separate 2 (Target: Self vs. Other) by 2 (Wrongness Admission: Admission vs. No Admission) factorial ANOVAs with each of the composite scores (and the “side” score) as the dependent variables. The first question asked whether scientists overestimate the negative reputational effects of failed replication efforts. We predicted that they do. A significant main effect of Target would provide evidence for this hypothesis, and that is exactly what we found for all composite and total scores (See Table 2 for relevant statistics).

**Table 2:** Means, Standard Deviations, Significance Tests, Effect Sizes, and Confidence Intervals for the Main Effects of Target (df = 1,277).

	Self *M*(*SD*)	Other *M*(*SD*)	*F*	*p*	η²_part_	90% CI
**Original Finding**	2.88(.71)	2.30(.66)	55.75	< .001	.17	.105-.232
**Suspicions of Other Work**	2.96(.78)	2.16(.69)	83.09	< .001	.23	.162-.298
**Scientific Reputation**	2.73(.71)	2.18(.68)	45.43	< .001	.14	.083-.204
**Social Reputation**	2.90(.64)	2.29(.73)	56.05	< .001	.17	.106-.233
**Total **	2.87(.61)	2.24(.59)	81.57	< .001	.23	.159-.294

The second question asked whether wrongness admission would result in more positive reputational outcomes for those whose work had failed to replicate. We predicted that it would. A significant main effect of Wrongness Admission would provide evidence for this hypothesis, and main effects for “original finding”, “scientific reputation”, and the “total” score were significant (See Table 3 for relevant statistics). The keen observer might notice that the aforementioned analyses included the Self and Other conditions, and that this question is better answered by the Other condition, alone. As such, we split the data by these two conditions and ran separate, one-way ANOVAs with Wrongness Admission as the independent variable. As seen in Table 3, when looking only at the Other condition all effects occurring in the whole sample had the same or a bigger effect size in the Other condition considered separately. This was not the case for the Self condition, as only the “original finding” effect remained significant.

**Table 3:** Means, Standard Deviations, Significance Tests, Effect Sizes, and Confidence Intervals for the Main Effects of Wrongness Admission (df = 1,277).

	Adm *M*(*SD*)	No Adm *M*(*SD*)	*F*	*p*	η²_part_	90% CI
**Combined**						
**Original Finding**	**2.38(.76)**	2.77(.68)	26.01	< .001	.09	.040-.141
**Suspicions of Other Work**	**2.54(.88)**	2.55(.79)	0.04	.841	.00	.000-.006
**Scientific Reputation**	**2.33(.78)**	2.57(.68)	9.76	.002	.03	.008-.076
**Social Reputation**	**2.58(.77)**	2.59(.73)	0.14	.706	.00	.000-.013
**Total**	**2.47(.72)**	2.62(.64)	5.76	.017	.20	.002-.056
**Other Only**						
**Original Finding**	**2.04(.60)**	2.55(.62)	25.92	< .001	.15	.072-.242
**Suspicions of Other Work**	**2.05(.63)**	2.27(.73)	3.54	.062	.02	.000-.079
**Scientific Reputation**	**2.03(.66)**	2.33(.67)	7.65	.006	.05	.008-.119
**Social Reputation**	**2.23(.71)**	2.34(.75)	0.92	.339	.01	.000-.045
**Total**	**2.09(.54)**	2.38(.61)	8.98	.003	.06	.007-.144
**Self Only**						
**Original Finding**	2.74(.74)	3.01(.65)	5.46	.021	.04	.003-.105
**Suspicions of Other Work**	3.04(.81)	2.86(.73)	1.82	.179	.01	.000-.062
**Scientific Reputation**	2.63(.79)	2.83(.60)	2.87	.092	.02	.000-.076
**Social Reputation**	2.93(.67)	2.88(.61)	0.24	.629	.00	.000-.031
**Total **	2.85(.67)	2.90(.54)	0.26	.612	.00	.000-.032

**Fig 2:**
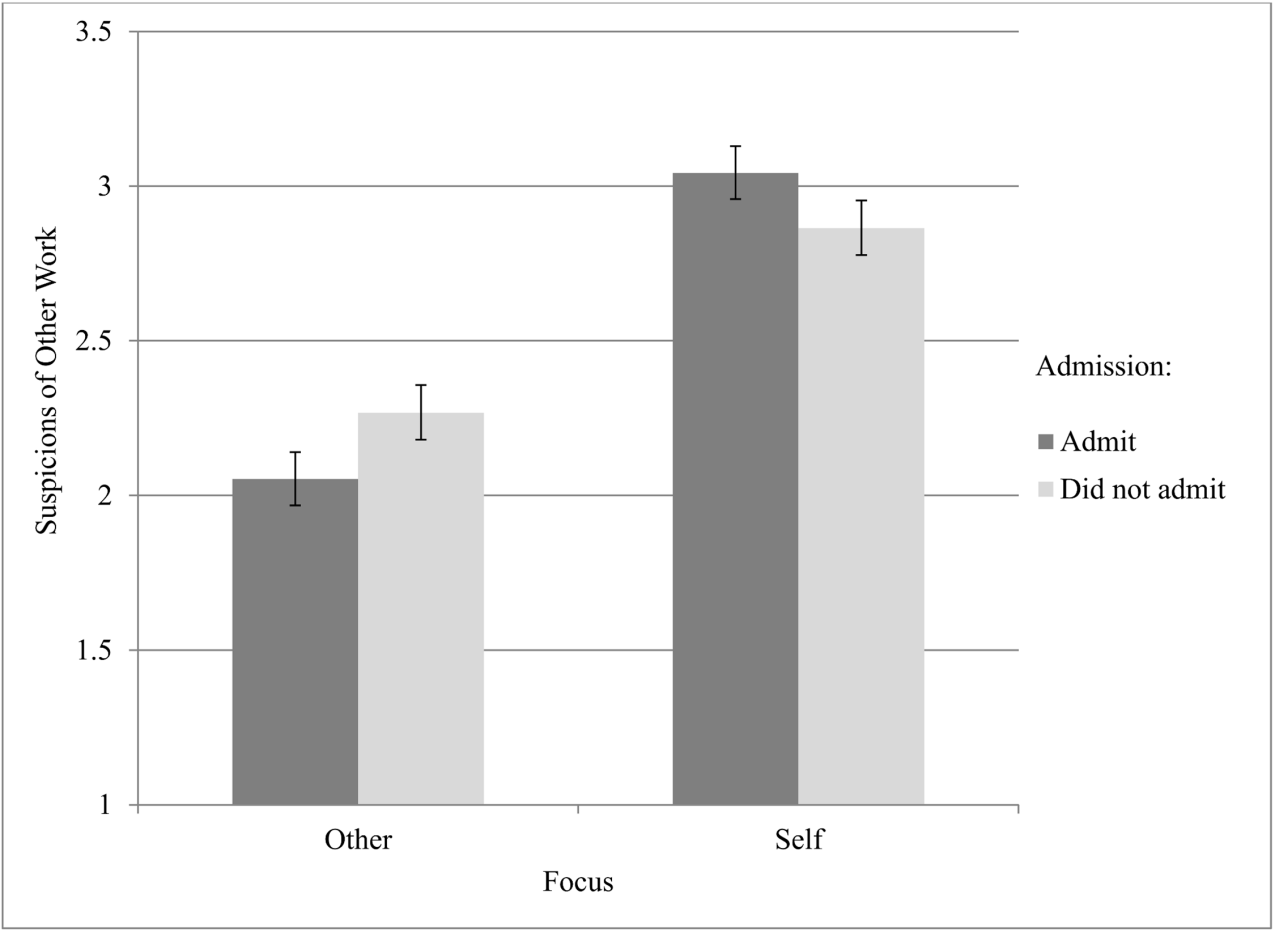
Means for the Focus x Admission interaction on “Suspicions of Other Work”.

The third question was whether or not scientists correctly estimate the reputational impact of admitting wrongness. We hypothesized that they do not. A significant Target x Wrongness Admission interaction would provide evidence for this hypothesis, and this was only the case for the “suspicions of other work” composite score (see Table 4). All other interaction effects were non-significant. The means for the significant interaction effect are displayed in Fig 2. As can be seen, this pattern supported our hypothesis that scientists believe that wrongness admission might lead to reputational suspicions of their work, but in reality, the opposite is true. It also comports with previous findings investigating non-scientists (Fetterman et al.). However, the non-significant interaction findings combined with the Self-only effects reported in Table 3 suggest that, in most cases, scientists do not see wrongness admission as either helpful or unhelpful for reputation, which makes it all the more curious as to why they do not admit.

**Table 4:** Significance Tests, Effect Sizes, and Confidence Intervals for the 2 x 2 Interactions (df = 1,277).

	*F*	*P*	η²_part_	90% CI
**Original Finding**	2.33	.128	.01	.000-.035
**Suspicions of Other Work**	5.10	.025	.02	.001-.052
**Scientific Reputation**	0.38	.539	.00	.000-.018
**Social Reputation**	1.06	.304	.00	.000-.025
**Total**	2.71	.101	.01	.000-.037

The final question was whether opinions about the “replication movement” change based on whether a failed replication effort is aimed at one’s own work versus the work of another person. We hypothesized that it would and in the direction that scientists would be less agreeable to the movement if their work was the target. A significant main effect of Target on “side” scores would provide evidence for this hypothesis, and this was the case, *F* (1,276) = 6.18, *p* = .01, η²_part_ = .02, 90% CI (.002,.058). Participants’ alignment with the replication movement were more toward the not important side if the replication efforts were aimed at one’s own work (*M* = 2.43, *SD* = .81) relative to the work of another scientist (*M* = 2.19, *SD* = .78). The other main effect, *F* (1,276) = 2.11, *p* = .15, and the interaction, *F* (1,276) = .61, *p* = .96, were not significant.

## Discussion

The purpose of this study was to apply recent work on public wrongness and wrongness admission to the recent replication debates in psychological science. In doing so, we hoped to provide insights, not only psychological scientists, but for all scientists in regards to dealing with controversial changes in their fields. We adapted previous study designs for this purpose. Utilizing a 2 (Target: Self vs. Other) by 2 (Wrongness Admission: Admission vs. No Admission) design, we were able to answer 4 questions. The first was whether or not scientists overestimate the negative reputational impact of failed replication efforts of their own work. Significant Target main effects on all measures suggest that they do. Relative to the Other conditions, those in the Self conditions anticipated a negative reputational impact.

Second, we wanted to know if wrongness admission would have a relatively more positive or negative impact on reputation. Significant Wrongness Admission effects on 3 of 5 reputation measures suggest that wrongness admission will not hurt one’s reputation, but may actually have a more positive effect. That is, relative to those in the No Admission conditions, those in the Admission conditions were less negative in their judgments.

Third, we also wanted to know if scientists were correct in their estimations about wrongness admission and reputation. Only 1 significant interaction (on “suspicions of other work”) suggests that scientists do not see wrongness admission as relatively helpful or hurtful. However, there is a hint that scientists have it backwards when it comes to creating suspicions for their other work. While they believed that admitting would be harmful, not admitting actually led others to become more suspicious.

Fourth and finally, we wanted to see if feelings toward the replication movement changed depending on whether one’s own work or the work of someone else was the target of replication efforts. A significant Target main effect on “side” scores suggests that the focus of replication efforts does matter. Relative to those in the Other condition, those in the Self condition had less favorable ratings toward the movement. These findings allowed us to apply our work to an ongoing debate, to answer some questions about the concerns of some researchers within this debate, and, perhaps, to provide insights for best practices for future interactions. This is the focus of the discussion.

### The Human Factors of Science

We like to think of science as a purely rational. However, scientists are human and often identify with their work. Therefore, it should not be controversial to suggest that emotions are involved in replication discussions. Adding to this inherently emotionally volatile situation, the recent increase in the use of social media and blogs by scientists [20] has allowed for instantaneous, unfiltered (i.e., lacking in editorial handling), and at times emotion-based commentary on research. Certainly social media has the potential to lead to many positive outcomes in science–among others, to create a more open science. To some, however, it seems as if this ease of communication is also leading to the public tar and feathering of scientists. Whether these assertions are true is up for debate, but we assume they are a part of many scientists’ subjective reality (cf.[3,21]). Indeed, when failed replications are discussed in the same paragraphs as QRPs, or even fraud, it is hard to separate the science from the scientist. QRPs and fraud are not about the science; they are about the scientist. We believe that these considerations are at least part of the reason that we find the overestimation effect that we do, here.

Even so, the current data suggests that while many are worried about how a failed replication would affect their reputation, it is probably not as bad as they think. Of course, the current data cannot provide evidence that there are no negative effects; just that the negative impact is overestimated. That said, everyone wants to be seen as competent and honest [8–9], but failed replications are a part of science. In fact, they are how science moves forward!

While we imply that the aforementioned effects may be exacerbated by social media, the data cannot directly speak to this. However, any one of a number of cognitive biases (e.g., [22]) may add support to this assumption and explain our findings. For example, it may be that a type of availability bias [23] or pluralistic ignorance [24] of which the more vocal and critical voices are leading individuals to judge current opinions as more negative than reality. As a result, it is easy to conflate discussions about direct replications with “witch-hunts” and overestimate the impact on one’s own reputation. Whatever the source may be, it is worth looking at the potential negative impact of social media in scientific conversations.

In addition, if the desire is to move science forward, scientists need to be able to acknowledge when they are wrong. Theories come and go, and scientists learn from their mistakes (if they can even be called “mistakes”). This is the point of science. However, holding on to faulty ideas flies in the face of the scientific method (Feynman, commencement speech). Even so, it often seems as if scientists have a hard time admitting wrongness. This seems doubly true when someone else fails to replicate a scientist’s findings. In some cases, this may be the proper response (e.g., the preponderance of the evidence is still in favor of the effect). Just as often, though, it is not. Further, we can now assert that, in most cases, admitting wrongness will have relatively fewer ill effects on one’s reputation than not admitting and it may be better for reputation. It could also be that wrongness admission repairs damage to reputation.

It may seem strange that others consider it less likely that QRPs, for example, were used when a scientist admits that they were wrong. However, it does make sense from the standpoint that wrongness admission seems to indicate honesty (e.g., in terms of a humble behavior [18]). Therefore, if one is honest in one domain, they are likely honest in other domains. Moreover, the refusal to admit might indicate to others that the original scientist is trying to cover something up. The lack of significance of most of the interactions in our study suggests that it even seems as if scientists might already realize this (though see below). Therefore, we can generally suggest that scientists admit they are wrong, but only when the evidence suggests they should.

Scientists are humans. As such, scientists are not immune to biases. It is easy to sit back and suggest that people are being irrational: That the original scientists are being defensive. However, our data suggest that one’s opinion about the replication movement is, at least weakly, associated with whether or not their work was the (imagined) target of a replication effort. These biases, then, might change the ways in which scientists respond to criticisms depending on the situation. If so, then it might be worth it to reread blog posts or Tweets from the perspective of the original scientists. Imagine how it might be construed if someone was proclaiming, for all your colleagues to see, that something you are proud of developing and publishing was a mistake or questionable. Then, perhaps, revise it. However, this is just our interpretation of the combination of overestimation and bias effects found in the current studies and we did not directly investigate the impact of harsh versus less harsh criticisms.

## Limitations & Future Work

There are a number of limitations to the current study that we would like to acknowledge. First, we utilized a completely convenience based sample. There are a couple reasons why this may be of particular interest to this study. First, while we did recruit from professional society listservs, a lot of our data was the result of Twitter buzz. Scientists on Twitter could be argued to be more active in the current debates and, indeed, more biased toward the replicator side of the spectrum. We have no way to validate this argument, but future work might look at what role social media use plays in the opinions of reputational impact, replications, and wrongness admission. It may also be that this bias explains the lack of interactions in our current findings, which tend to be present in other populations (e.g., Fetterman et al.). For example, participants may have been indicating what should be thought versus what they would really think (i.e., in the Self conditions) or they may just have systematically different opinions which value wrongness admission.

The latter consideration should also render the scale labels used as less meaningful and a relativistic interpretation of the means as more informative. Further to this point, we did not have a neutral condition, so we cannot make any conclusions about the general feelings about failed replications. Future work should look into this. A second problem with the convenience sample is that we did not get a particularly large sample of the field and the sample was primarily composed of psychological scientists. There is nothing we could do about this particularly (we cannot dip back into the pool without overlapping samples), but future studies might want to utilize more influential connections to get a more representative sample and more participants from outside psychology.

The aforementioned issue about “ought” versus “actual” views may also be a problem that arises from our scenario-based methodology. While scenario-based methodologies are relatively common in social psychology, there are any numbers of biases that may influence a non-real situation. Suffice it to suggest that we cannot be sure what anyone’s *real* reactions would be without them having experienced the actual scenario. In fact, we would be willing to bet that our effect sizes would have been much larger–and many of the non-significant ones been significant–had we actually manipulated the situation or surveyed individuals whose work had been targeted. However, one can see the ethical dilemma in the former and the dearth (and apprehension) of individuals in the latter. Therefore, future work might find a way to get at these populations, if needed. We would like to note, however, that there is plenty of precedent for using scenario-based studies when direct manipulations cannot be completed. In fact, recent work on meta-science has employed these methods (e.g., [25]). One benefit of our sample, however, is that it was comprised of published scientists, as opposed to lay-persons.

In addition, there are clearly more than these two options (admitting versus refusing) for responding to failed replication efforts. For example, one might simply say “these diverging results are interesting”. Anecdotally, these responses seem rare. In any case, it was our goal to highlight the potential downsides of refusing to admit and the upsides of admitting wrongness. In some cases, we believe, this third option could be interpreted as a humble acceptance of the replication efforts and in other cases–when a replication effort is thorough–it could be interpreted as humble rejection of the replication efforts. Future research should quantify the differences in such responses, but it was our goal to investigate responses that minimize alternative interpretations.

Finally, we recognize that the current methods may not represent the totality of the reputational situation. Of course, no study can account for every variable that might play a role in the situation. As such, we were really looking for the core finding. Future research should look at nuances to our study. For example, one might take into consideration the previous work of the scientist in question. Is it replicable? Has their work been under scrutiny before? One could also look at the target scientist’s tenure in the field. Are they a professor or a junior scientist? Will a junior scientist’s work being targeted lead to an inability to get a job? Future work might even use the “side” measure as a moderator in such reputational considerations. There are a number of questions that can be asked and we hope that our work will spark a number of lines of intriguing research.

## Conclusions

We would like to reiterate again that we believe our effect sizes to be *under*estimates of the true effect and that our small effects signify large implications. They suggest that the current atmosphere is creating worry. In turn, this worry may undermine the *trust* that our science relies on. Without this trust, we cannot survive as a field. We have all made mistakes and we are all learning as we go. As such, there is no reason to single anyone out and unjustly blame a scientist for failed replications. Indeed, we do not see this as the goal of the replication movement. Even so, the goal of a movement is not always as important as the perception of its motives. As such, if scientists fear for their reputations, then the replication movement may close, rather than open, our science. Open science requires trust on both sides and the felt ability to admit wrongness. Being wrong is a part of science. If scientists equate wrongness admission with an admission to sloppy research practices, or even fraud, it seems logical that scientists will be very hesitant to do so.

Social media is an important new component to how science is communicated. It allows people to discuss science more openly and allows for much needed skepticism. However, it also allows for unfiltered and, often times, irreversible comments. These comments may be viewed as hostile, if not carefully constructed. Perhaps partially due to these issues, some scientists have grown fearful about their reputations being tarnished by failed replication efforts. They have perceived the movement as “bullying” [3]. As a result, they have also been resistant to replication efforts and to admitting that previous findings might be wrong. We, indeed, found that scientists do fear for their reputation and overestimate the negative reputational impact of failed-replications. Second, we found that admitting wrongness is less harmful to one’s reputation than not admitting and it may actually help it. Indeed, not admitting can only hurt one’s reputation. In light of these findings, it is our perspective that we all need to be more careful about how we communicate and to keep an open, trustful mind as science evolves. These findings and suggestions are not only for psychologists: They apply to all sciences.

## Supporting Information

S1 File Redacted Data (CSV)

S2 File Supplementary File Containing Violin Plots for the Significant Effects. (DOCX)
