# Supplementary material for: The Reputational Consequences of Failed Replications and Wrongness Admission among Scientists
Source: PLoS One. 2015 Dec 9;10(12):e0143723. doi: 10.1371/journal.pone.0143723 (PMC4674057; doi:10.1371/journal.pone.0143723)
Supplement: S2 File — (DOCX) [file pone.0143723.s002.docx]

**Significant Main Effects for *Focus***
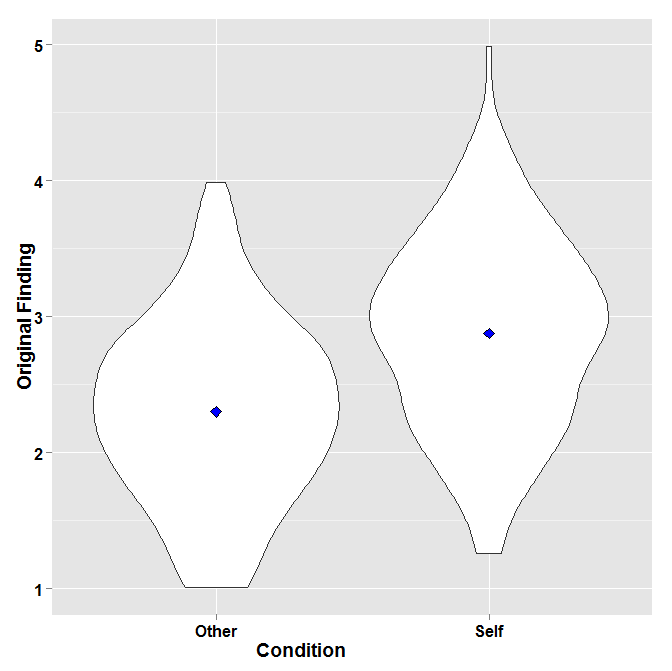


Note: Higher score reflects more negative reputational ratings. Blue dot represents the mean.


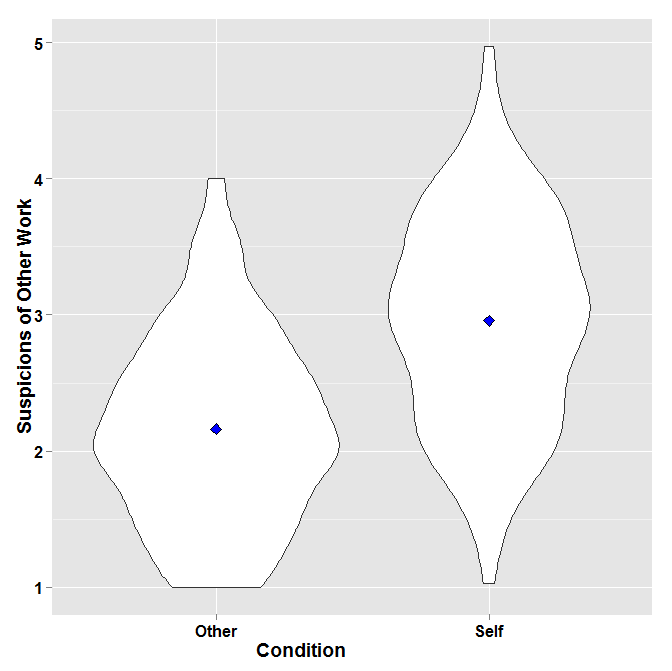
 Note: Higher score reflects more negative reputational ratings. Blue dot represents the mean.


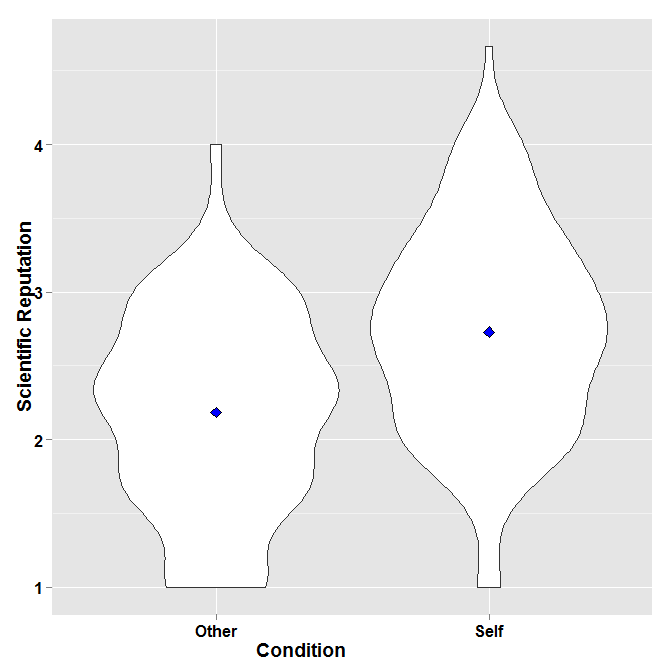
 Note: Higher score reflects more negative reputational ratings. Blue dot represents the mean.


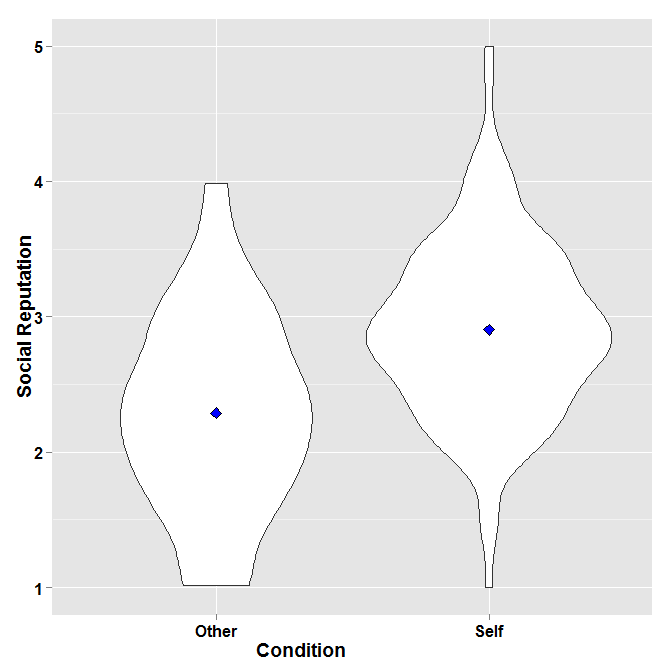
 Note: Higher score reflects more negative reputational ratings. Blue dot represents the mean.


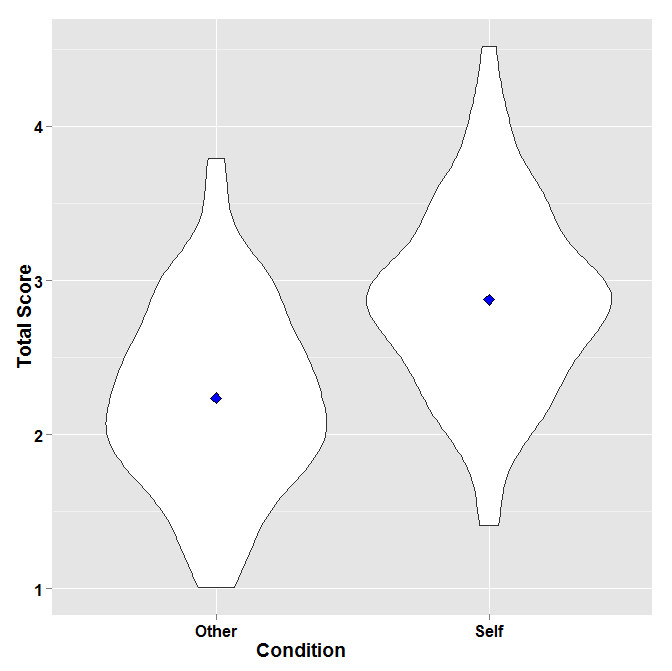
 Note: Higher score reflects more negative reputational ratings. Blue dot represents the mean.

**Significant Main Effects for *Admission***
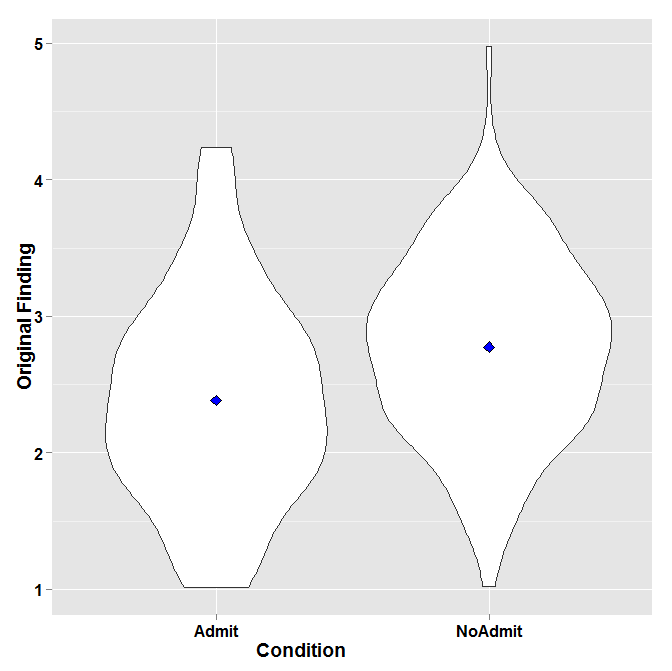
 Note: Higher score reflects more negative reputational ratings. Blue dot represents the mean.


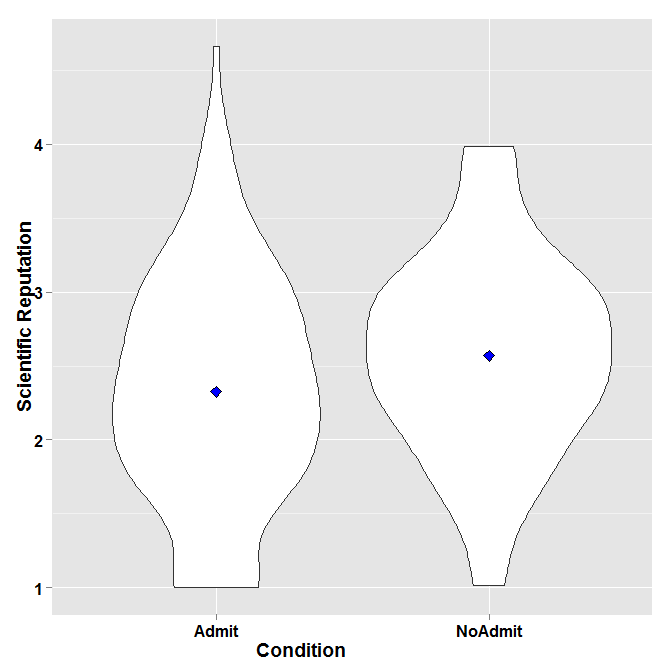
 Note: Higher score reflects more negative reputational ratings. Blue dot represents the mean.
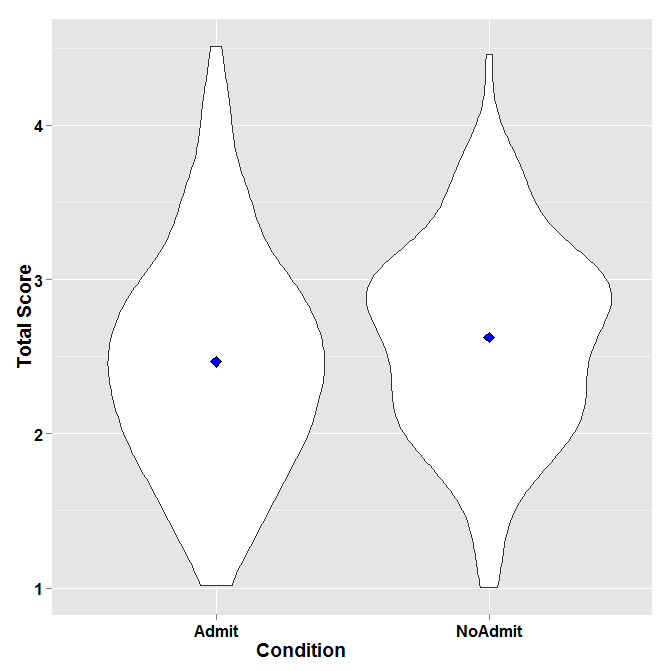


Note: Higher score reflects more negative reputational ratings. Blue dot represents the mean.

**Significant *Self* x *Admission* Interaction on Suspicions of Other Work**


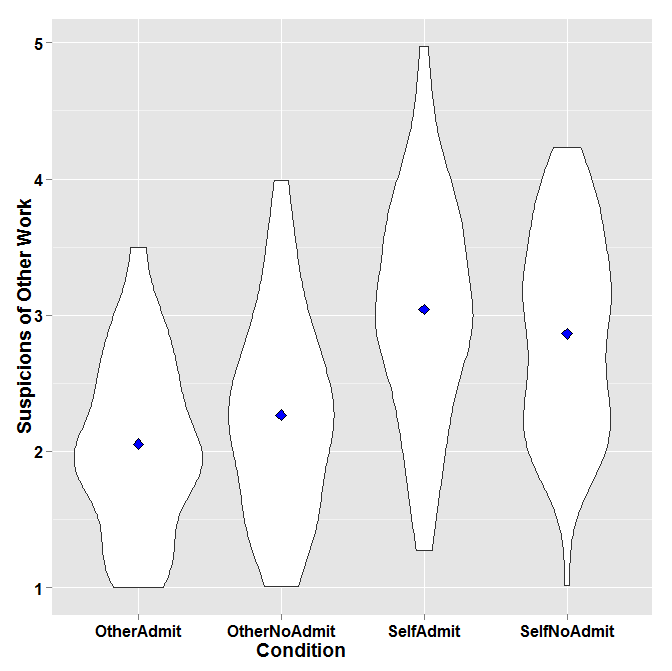
 Note: Higher score reflects more negative reputational ratings. Blue dot represents the mean.

Significant Main Effect of Focus on *Side*
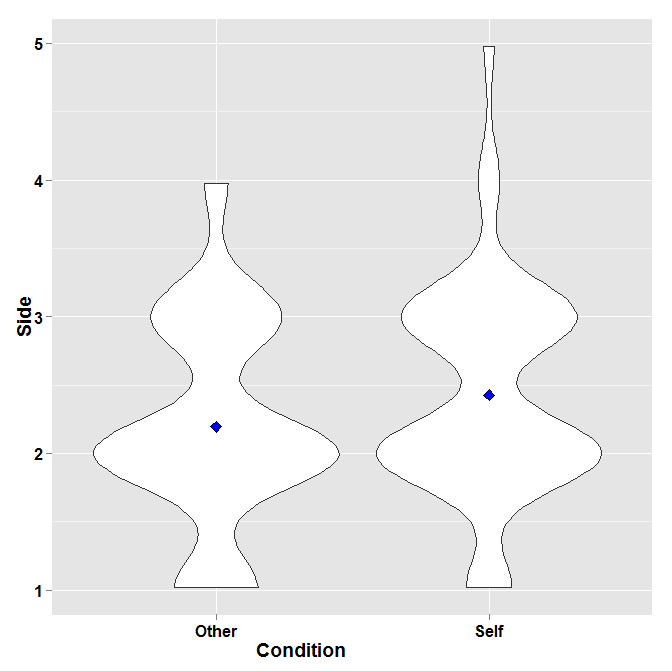
 Note: Higher score reflects less endorsement of the replication movement. Blue dot represents the mean.
